# A Coordinated Public Health Laboratory Response to COVID-19 in Mali

**DOI:** 10.3389/fitd.2021.788616

**Published:** 2022-01-30

**Authors:** Bassirou Diarra, Amadou Kone, Ibrehima Guindo, Sidy Bane, Lassina Doumbia, Lassana Timbine, Dramane Diallo, Moumine Sanogo, Antieme Combo Georges Togo, Tenin Aminatou Coulibaly, Fatimata Amath Diallo, Mahamadou Kone, Anou Moise Somboro, Josue Togo, Mariam Coulibaly, Fatoumata A. Camara, Gagni Coulibaly, Hawa Boukary Diarra, Mahamane Talphi Diakite, Mohamed Abdou, Amadou Somboro, Oumar Dolo, Oumou Ousmane Maiga, Daouda Keita, Youssouf Coulibaly, Boureima Degoga, Hawa MBaye Drame, Mariame Sow, Mariam Goumane, Fah Gaoussou Traore, Kadidia Kone, Fanta Sanogo, Ibrahima B. Diallo, Larissa Denou, Yeya Sadio Sarro, Katy Shaw-Saliba, Chuen-Yen Lau, Aaron Neal, Idrissa Sow, Bourema Kouriba, Ousmane Koita, Mahamadou Diakite, Akory Ag Iknane, Seydou Doumbia

**Affiliations:** 1 University Clinical Research Center (UCRC), University of Sciences, Techniques and Technologies of Bamako (USTTB), Bamako, Mali,; 2 Laboratory and Biomedical Research’s Department, National Institute of Public Health (INSP), Bamako, Mali,; 3 Laboratory for Applied Molecular Biology (LBMA), University of Sciences, Techniques and Technologies of Bamako (USTTB), Bamako, Mali,; 4 The Charles Mérieux Infectiology Center (CICM) of Bamako, Ministry of Health and Social Affairs, Bamako, Mali,; 5 Collaborative Clinical Research Branch, Division of Clinical Research, National Institute of Allergy and Infectious Diseases (NIAID)/National Institutes of Health (NIH), Bethesda, MD, United States

**Keywords:** COVID-19, laboratory responses, UCRC, Mali, sustainability

## Abstract

Ability to rapidly and accurate diagnose pathogens during disease outbreaks is essential for public health. Diagnosis depends largely on laboratory capacity, which can be challenging in resource limited settings. We report Mali’s experience involving four research laboratories in response to the COVID-19 pandemic. This coordinated effort leveraged the emerging infectious pathogens diagnostic capacity and partnerships built from the 2014/2015 Ebola outbreak. Since Mali’s first two COVID-19 cases in March 2020, 349,292 suspected cases were tested in the four Bamako laboratories as of July 31, 2021. Laboratory operation, safety considerations, diagnostic assays, and challenges are described herein from the perspective of a participating laboratory, the Mali University Clinical Research Center (UCRC). We also highlight additional roles of the UCRC laboratory in the COVID-19 response, including roll out of vaccination and research efforts. Mali’s readiness to detect the index cases early in the epidemic and continued response to the COVID-19 pandemic highlight the need for strengthening the critical role and capacity of clinical research laboratories for response to emerging infectious disease epidemics in Africa.

## 1 INTRODUCTION

COVID-19 was first recognized in December 2019, though studies suggest it was circulating earlier. The pandemic is still ongoing 1.5 years later and epicenters have shifted with multiple waves and variants. There is considerable concern that low vaccination rates in Africa and circulating variants of concern will impair COVID-19 control. Rapid confirmation of COVID-19 cases is important not only for patient management, but also for limiting human-to-human transmission through immediate case isolation and contact tracing. In West Africa, suspected viral infectious disease specimens have generally been sent to the WHO Reference Laboratory at Pasteur Institute in Dakar, Senegal. The need to transfer specimens from outlying countries, including Mali, and assay turnaround time hinder patient care and public health efforts. COVID-19 has additionally challenged this already suboptimal laboratory mechanism with high suspected case numbers, extended delays related to import and export regulations, complicated shipping logistics, air travel restrictions, and border closures, making use of a central referral laboratory for decision making impractical (1, 2).

Based on experience from prior disease outbreaks including the 2014 Ebola epidemic, Mali developed a proactive approach for detection, containment, and management of COVID-19 infected cases in February 2020 (3). Ramped up capacity enabled identification of the first cases in two travelers from France on 25 March 2021 by laboratories in Mali (4). However, capacity was still limited, and many clinicians relied on clinical presentation and imaging for diagnosis during the first wave (April-June 2020). While clinical presentation and imaging (chest x-ray and CT) are very helpful, laboratory testingLISAand rapid) is essential for pathogen confirmation. reliedchest X-rays and CT scans for diagnosis (5). Diagnostic capacity was thus further expanded during the pandemic. Here, we describe the pivotal role played by the four public health research laboratories in the Malian National COVID-19 response. Preparedness for an outbreak response, workflow from sample reception to results communication to the Malian National COVID-19 response committee, and continued evolution of the laboratory’s role in the COVID-19 response is shared as a model for other laboratories in low resource settings.

## 2 PUBLIC HEALTH GENERAL AND LABORATORY RESPONSES TO COVID-19 IN MALI

### 2.1 Laboratories Performing SARS-CoV-2 Testing

The Malian ministry of health (MoH) designated four laboratories associated with research institutions working on infectious diseases and outfitted with biosafety containment levels from 2 to 3+ for SARS-CoV-2 diagnostic and confirmatory testing: 1) the National Institute of Public Health (INSP); 2) the University Clinical Research Center (UCRC); 3) the Charles Mérieux Infectiology Center (CICM); and 4) the Laboratory for Applied Molecular Biology (LBMA). Based on experience gained during the 2014/2015 Ebola outbreak response, UCRC was the first COVID-19 referral laboratory selected by Malian Ministry of Health (3) in February 2020 and was the only laboratory designated prior to detection of Mali’s first SARS-CoV-2 infection. Projected increases in cases engendered selection of the three additional laboratories based on their available biosafety level, capacity to perform necessary assays and anticipated access to reagents.

#### 2.1.1 National Institute of Public Health (INSP)

INSP currently serves as the national referral laboratory for disease surveillance. INSP is a member of the WHO-AFRO laboratory network, and regularly participates in the proficiency testing program and trainings on emerging diseases. To address COVID-19, two Biosafety Level 2 (BSL-2) laboratories were established - one dedicated to nucleic acid extraction in a certified class 2 biosafety cabinet (BSC) and the second dedicated to PCR master mix preparation and amplification.

**FIGURE 1 |:**
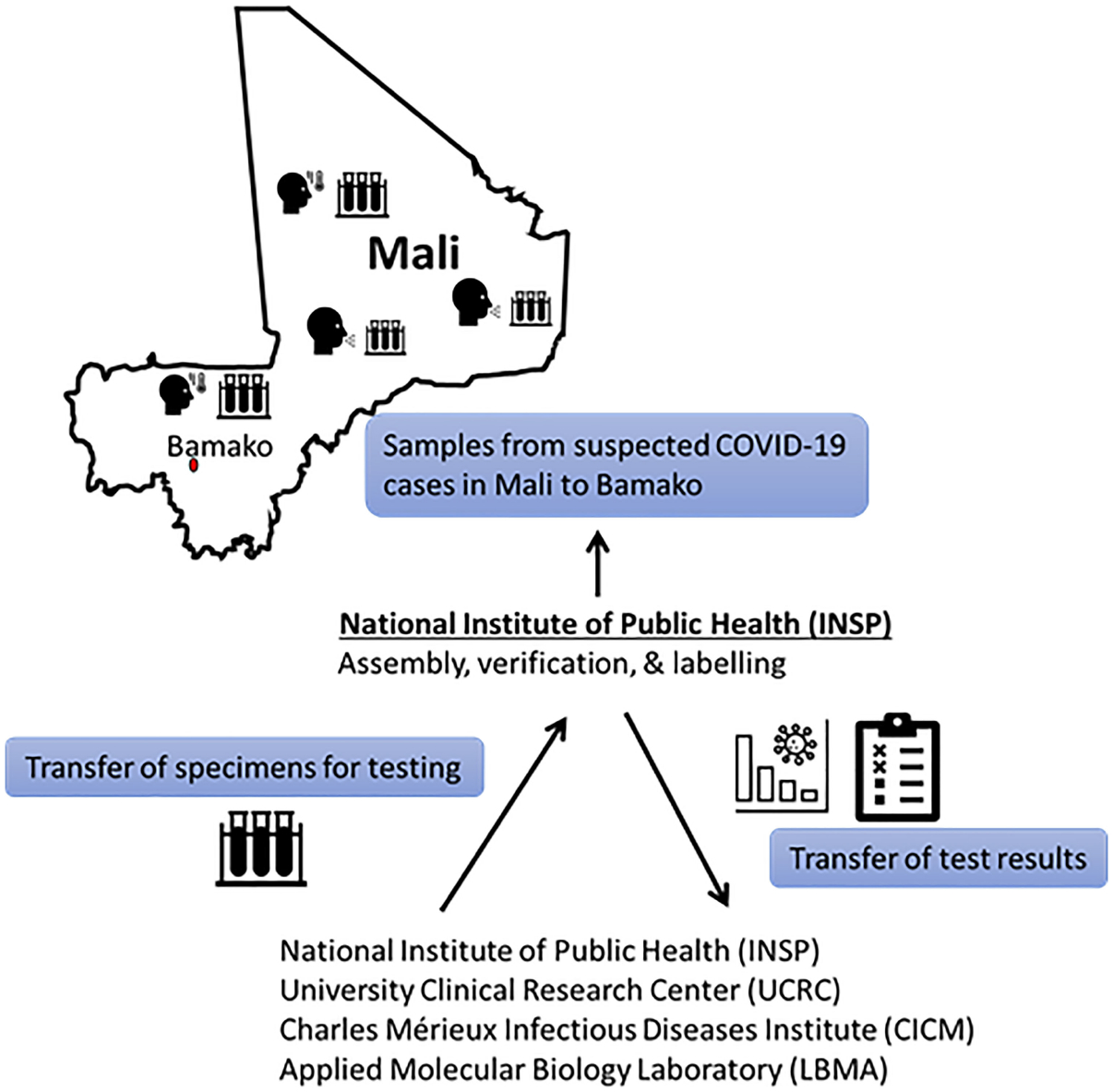
Initial flow of Malian COVID-19 suspected samples and reporting until 15 December 2020. After 15 Dec 2020, specimens could be sent directly to the processing laboratory without being curated by INSP. Processing laboratory communicates results to care providers and INSP communicates results to National database.

INSP collaborates with other laboratories at central and district levels to ensure proper specimen collection from suspected infectious disease cases and efficient specimen transport. Until December 15^th^, 2020, INSP received all specimens from suspected COVID-19 cases collected in Mali. Suspected cases included those with consistent symptoms, close contact with known cases, or recent travel. Samples were then cataloged, labeled, and dispatched to the other three SARS-CoV-2 diagnostic laboratories (Figure 1). Results were typically returned within 1 week. Although INSP still provides central coordination of the COVID-19 laboratory response, samples are now routed directly to specific laboratories to reduce turnaround-time, which is currently less than 24 hours. Results are reported back to INSP and the care provider or patient simultaneously.

#### 2.1.2 University Clinical Research Center (UCRC)

Located at the Faculty of Medicine, Pharmacy and Dentistry of the University of Sciences, Techniques and Technologies of Bamako (USTTB), UCRC is a joint initiative between the government of Mali and the United States National Institutes of Health (NIH) to facilitate clinical research and support national disease surveillance systems through laboratory diagnostic capacity (4). The center has immunology, molecular biology, and microbiology Biosafety Level-3 laboratories certified by WHO and College of American Pathologists (CAP). UCRC played a critical role in laboratory testing during the West African Ebola outbreak of 2014/2015 (3). Based on those experiences, and in collaboration with the Laboratory of Virology of the NIH Rocky Mountain Laboratory, Hamilton, Montana (USA), UCRC developed and validated a SARS-CoV-2 RT-PCR assay in February 2020 before initial cases were reported in Africa. Validation was done using positive and negative controls based on the originally published sequence provided by US partners. Additionally, UCRC was able to secure other reagents and supplies required for SARS-CoV-2 diagnostic testing through this partnership. Thus, UCRC was the first laboratory designated to perform SARS-CoV-2 testing in Mali. Virus inactivation is performed in the BSL-3+ laboratory. Subsequent RNA extraction, RT-PCR, and result confirmation is performed at the BSL-2 Molecular Core laboratory.

#### 2.1.3 The Charles Mérieux Infectiology Center (CICM)

Currently, the only medical laboratory accredited International Standard Organization (ISO 15189) within the four, CICM’s mission is to participate in health service through training, research, and capacity building in the field of diagnostics. The CICM encompasses a medical analysis laboratory with bacteriology-virology, hematology, serology, parasitology, biochemistry, and molecular biology capacities. Its research activities focus on infectious diseases including tuberculosis, HIV/AIDS, hepatitis B and C, in partnership with national structures, including hospitals and other patient care centers, and external partners throughout Mali. A major function of CICM is assisting the INSP with culture and drug susceptibility testing for multi-drug resistant Tuberculosis in Mali. During the COVID-19 pandemic, in addition to their fixed container BSL-3 in Bamako, CICM deployed a mobile BSL-3 laboratory. This mobile laboratory could be moved to any region of the country within 1 day by plane or car, though it was mostly used in the northern (Timbouctou) and central (Mopti) regions. CICM was assisted by *Fondation Mérieux* and the German government to develop and deploy its SARS-CoV-2 diagnostic capacity.

#### 2.1.4 The Laboratory for Applied Molecular Biology (LBMA)

LBMA is located at the Faculty of Sciences and Techniques of USTTB and is an international center of excellence for training and research in health and agriculture. The LBMA encompasses four research units including a parasitology unit, a plant/animal biotechnology unit, a virology unit, and a clinical biology unit. It has a BSL2 laboratory set up specifically for outbreak response. LBMA also supports national surveillance for zoonotic diseases in Mali and collaborates with several research institutions at the national and international levels. LBMA has partnered with the Research Institute for Development in France and the US CDC, which helped secure COVID-19 diagnostic supplies.

### 2.2 Laboratory Activities

Mali recognized early on that minimizing COVID-19 associated morbidity and mortality would require strong coordination amongst all components of the public health response. Thus, public health authorities sought a harmonized approach to development of case detection strategies, contact tracing systems and infection control measures. The laboratories played critical roles in specimen collection, management and reporting aspects of the COVID-19 response.

#### 2.2.1 Clinical Specimens

Specimens were obtained under public health surveillance and not as part of human subjects’ research. Nasopharyngeal (NP) or Oropharyngeal (OP) swabs were collected by trained medical personnel at referral health centers or treatment centers using sterile synthetic fiber swabs with plastic or wire shafts pre-wetted in Viral Transport Medium (VTM). If both NP and OP swabs were collected, they were combined in a single tube to maximize test sensitivity and limit resource use. Until 15 December 2020, all samples were assembled at the INSP, and then dispatched to the other three laboratories for testing based on laboratory capacity; after 15 December 2020, samples could be sent directly to the testing laboratory. Capacity has continued to expand during the pandemic, with UCRC able to process up to 700 specimens in a day.

#### 2.2.2 Virus Inactivation, Nucleic Acid Extraction and Quantitative Reverse Transcription Polymerase Chain Reaction (qRT-PCR) Assays

Laboratories established procedures to effectively perform all necessary steps of SARS-CoV-2 detection. Sample inactivation was performed under BSL-2 in INSP and LBMA laboratories, and in a BSL-3+ laboratory at CICM and UCRC. Inactivated samples were transferred to the BSL-2 molecular biology laboratory where RNA extraction was performed according to manufacturer’s protocols (6). RT-PCR assays were performed using several kits that employ a variety of SARS-CoV-2 RNA gene targets, including envelope (*E*), nucleocapsid (*N*), spike (*S*), RNA-dependent RNA polymerase (*RdRp*), and *ORF1* genes (6–9). While Mali’s ongoing collaborations supported an uninterrupted supply of testing materials, variation in kits occurred as reagents were supplied at different times though the US National Institutes of Health, Jack Ma Foundation, Mali government and UNICEF.

For public health purposes, results were released to the country COVID-19 response coordinator after confirming positive results in the performing laboratory. The response coordinator still reports to the Ministry of Health and then releases a daily report to inform the community about the COVID-19 situation in Mali. Reporting typically occur within 6–20 hours after sample receipt but definitely within 24 hours (Figure 1).

### 2.3 Laboratory Findings and Patient Characteristics

#### 2.3.1 Number of Samples Received and Tested

**FIGURE 2 |:**
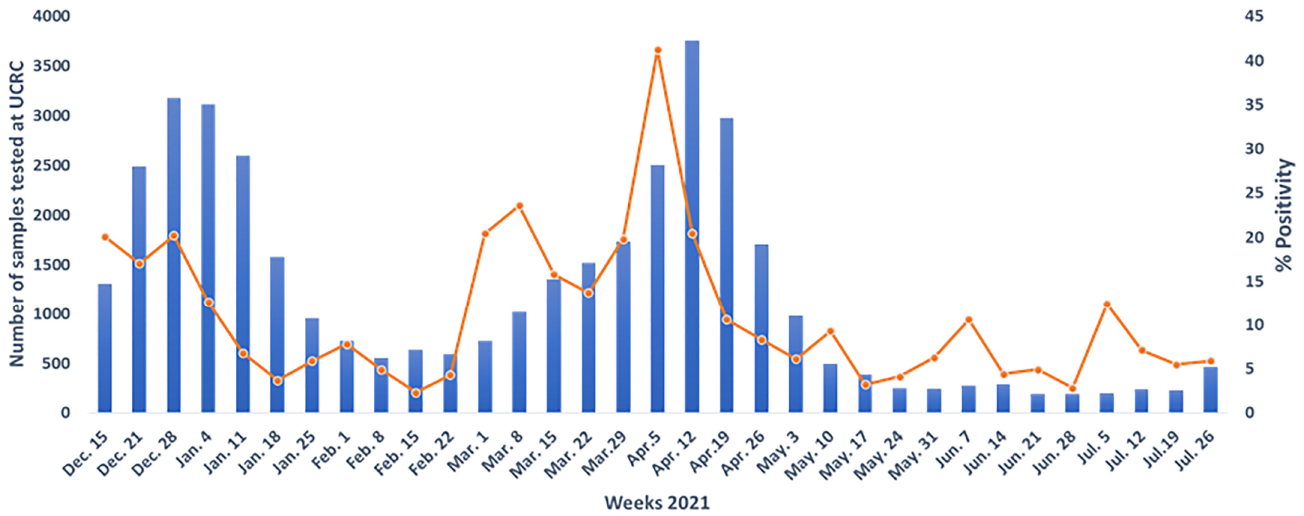
The trajectory of specimens tested and the positivity rate during 2021 at University Clinical Research center (UCRC).

While INSP, UCRC, and LBMA are all based in Mali’s capital of Bamako, the mobile diagnostic laboratory CICM performed testing in Bamako, Tombouctou (northern Mali), and Mopti (central Mali) depending on local case rates. Cases in Tombouctou and Mopti were elevated in May-June 2020 and June-July 2020 respectively. Nonetheless, Bamako has remained the epicenter Mali’s outbreak. The four designated Malian laboratories have cumulatively tested 349,292 suspected samples between February 25^th^, 2020, and July 31^st^, 2021. None had to turn specimens away due to lack of testing capacity (10). Of the specimens tested, 14,587 (4.18%) were positive. In addition, from February 25^th^ to December 9^th^, 2020, UCRC received 29 448 (suspected + patients under treatment) from INSP and found 2, 630 positives. Figure 2 shows the trajectory of specimens tested and the positivity rate during 2021 at UCRC.

#### 2.3.2 Characteristics of SARS-CoV-2 Positive Patients

Amongst 14,587 positive cases as of July 31^st^ 2021, 33% were female and 67% were male (10). 30–34 year-olds constituted the most represented age group for both genders, with a mean age of 39.7 ± 17.5 years (10). The total death rate among positive patients was 3.65% (533/14,587).

#### 2.3.3 COVID Vaccine Administration

During the pandemic, the UCRC and INSP laboratories expanded their role to include provision of COVID vaccinations. This was done to facilitate the public health goal of making vaccines as accessible as possible. Mali has largely been dependent on donations by COVAX. The first shipment in March 2021 included AstraZeneca and a recent shipment in August 2021 included second doses of AstraZeneca and Johnson and Johnson. Potential vaccinees no longer need to travel into the city center or to a special site, which could require significant time and financial resources, especially when the vaccination regime requires two doses. There are several Health Care Centers or Hospitals, in Bamako or in many other cities for vaccine administration. Furthermore, the public health laboratories can collect samples from vaccinees to generate seroprevalence data, which was used by policy makers to guide additional planning for vaccine deployment. The laboratories were also able to follow vaccine recipients for serious adverse events, none of which were seen.

## 3 DISCUSSION

### 3.1 Lessons Learnt Learned From the COVID-19 Laboratory Response

#### 3.1.1 Strengths and Weaknesses

With the large sample throughput, false positive and false negative results would be expected and likely occurred. When concerned about a false positive result, repeat or follow up testing can be performed. Viral culture could also be included in the diagnostic algorithm to determine if the isolated virus is viable (6), but this is much more labor and resource intensive and not readily available in Mali. The huge number of suspected cases associated with the COVID-19 pandemic rendered culturing of all viruses infeasible.

Suspected case numbers have also meant that Mali is unable to systematically evaluate all case contacts for SARS-CoV-2 infection. Case rates may thus be underestimated. Other countries in the region have the same constraint. While Egypt was the first African country to report a COVID-19 case on 14 February 2020, cases were subsequently reported from every African country (11). Adequate community testing has been hindered by limited infrastructure and test kit availability, in part due to lockdowns and supply chain interruptions across Europe, Asia and America (11). Also, returning of results to patients or family members was occasionally not possible. Contact information may have been inaccurate, patients may have relocated, or stigma may have hindered follow-up.

A last overarching challenge commonly faced by resource limited settings was lack of national funding for the referral laboratories. The Malian government relied on support from partners and donors to establish the necessary capacity. While Mali was able to establish laboratory capacity for COVID-19, this model may not be sustainable.

#### 3.1.2 Opportunities and Threats

Challenges present an opportunity to improve. Our referral laboratories are continuously evaluating strategies for optimizing their performance, as well as how to integrate with other components of the public health infrastructure. Administration of COVID vaccines by UCRC and INSP as part of the emergency response represented a major paradigm shift.

Although it is not feasible to do viral cultures for every positive specimen, there are situations in which this would be useful. UCRC is currently establishing this capacity in its BSL-3+ facility. It is hoped that other laboratories in Mali will also develop this capacity. The complementary ability to do large scale serologic testing for antibodies is also being implemented with the goal of monitoring the epidemiology of SARS-CoV-2 exposure within the Malian population. Sero-prevalence data will enable determination of more accurate case fatality rates and identification of high-risk groups, allowing limited resources to be targeted where they will have greatest impact.

Partnerships with private laboratories are also under consideration. Some private facilities have capabilities unavailable through the government laboratories, such as ISO 15189 and molecular diagnostic facilities. Engagement of additional laboratories can also increase testing throughput. Thus public-private partnerships have the potential to enhance the ability to mount a quick pandemic response. Within laboratories, further improvement of turnaround time, ideally to providing results during the initial testing visit, would improve ability to return and act upon them.

From the policy standpoint, reliable national funding for maintenance of critical public health resources is also a goal. This would facilitate maintenance of physical laboratory infrastructure as well as human capital. It would be helpful to have referral laboratories strategically located throughout the country. All the laboratories described in this paper are in Bamako, the capital city. This is largely because Bamako is the most populated area of Mali and thus was able to support original establishment of the laboratories. Although Bamako is the epicenter of the current Malian COVID-19 outbreak, COVID-19 epidemiology could change, and future outbreaks may be concentrated elsewhere.

### 3.2 Outlook

UCRC, CICM and LBMA will continue to support public health activities as needed. Although established as research facilities, these laboratories represent capacity that can be rapidly activated as infections emerge. Between outbreaks, capacity is maintained at a basal level through research endeavors that focus on locally relevant diseases. Referral laboratory staff are thus given the opportunity for training and acquisition of new skills (12). This model ensures that expertise and infrastructure development are continuously supported. During epidemics, the research at public health laboratories may offer unique capabilities, such as deep sequencing or virus characterization, which would complement traditional public health laboratory functions.

Ministry of Health decision makers will also explore the possibility of establishing activable referral laboratory capacity in regions outside of Bamako. Strategic placement of diagnostic facilities will enable more rapid returning of results, which would in turn facilitate a more efficient public health response. As outbreak epicenters are unpredictable, enhancement of mobile lab capacity is also being considered. This would allow critical services to be rapidly deployed to areas without existing facilities. Improved laboratory accessibility could improve not only turnaround time but may also increase testing uptake with corresponding improvements in epidemiologic characterization.

## 4 CONCLUSION AND POLICY RECOMMENDATIONS

Early detection of infectious diseases including COVID-19 and containment present challenges for resource constrained countries such as Mali. Mali continues to make strides in developing a laboratory infrastructure that will facilitate an efficient and effective response to epidemics, consistent with the WHO International Health Regulations, which provide an overarching legal framework that defines countries’ rights and obligations in handling public health events and emergencies that have the potential to cross borders (13). In this paper, we have shared the role of Mali’s referral laboratories in responding to the COVID-19 outbreak as a means of highlighting important considerations in building laboratory capacity. We have also identified key components of developing laboratory capacity in resource limited settings (Box 1).

In-country laboratory diagnostic capacity is critical for responding to outbreaks of emerging and re-emerging infectious diseases such as the COVID-19. Rapid and reliable test results support clinical management and epidemiologic investigations. Our work demonstrates the infectious disease laboratory’s critical role in pandemic preparedness and response. With coordination and partnerships, an effective public health response can be conducted in a resource limited setting during an outbreak. Of note, the SARS-CoV-2 diagnostic activities described herein were run by a local team in Mali, improving sustainability. This response strategy can be applied in other resource limited setting to establish sustainable laboratory capacity. Building diagnostic capacity in resources-limited-setting such as Africa will support global preparedness for future pandemics.

## Data Availability

The raw data supporting the conclusions of this article will be made available by the authors, without undue reservation.

## BOX 1 | Recommendations for establishing laboratory capacity in low-resource settings.

Assess existing capacity and identify gaps

Develop a scalable laboratory model

Partner with international organizations to develop human and physical infrastructure

Consider public-private partnerships to address resource needs

Identify and prioritize strategic geographic locations for laboratories

Secure national government support to ensure sustainability
